# Leveraging Barangay Health Workers to Strengthen Geriatric Care in an Aging Philippines

**DOI:** 10.1002/agm2.70115

**Published:** 2026-10-10

**Authors:** Darin Jan C. Tindowen

**Affiliations:** ^1^ Commission on Higher Education—Region 2 Tuguegarao City Philippines

## Abstract

Population aging in the Philippines requires innovative, community‐based approaches to strengthen geriatric care delivery. This article highlights the potential of Barangay Health Workers as frontline partners in promoting healthy aging through early detection, chronic disease monitoring, and coordinated referral services. Expanding BHW roles through standardized training and policy support can address gaps in geriatric healthcare access. A strengthened community health workforce can contribute to an age‐responsive and sustainable Philippine healthcare system.

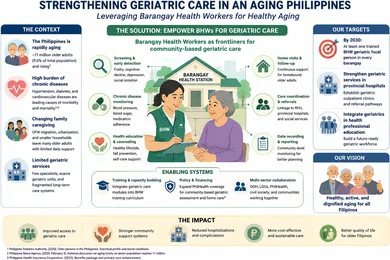

Dear Editor,

1

The Philippines is undergoing a rapid demographic transition toward population aging. National statistics indicate that older adults aged 60 years and above now comprise approximately 9%–10% of the population, or about 11 million individuals, with projections indicating continued growth in the coming decades [1, 2]. This shift places the country on the threshold of an aging society and presents urgent implications for health system readiness.

The aging population is accompanied by a rising burden of non‐communicable diseases and functional decline. Evidence shows that cardiovascular diseases, diabetes, and chronic respiratory conditions remain leading causes of morbidity and mortality among older Filipinos, contributing significantly to healthcare utilization and expenditures [2]. In addition, global evidence highlights that multimorbidity and frailty increase substantially with age, requiring continuous, coordinated, and community‐based care models rather than episodic facility‐based services [3].

Despite these trends, geriatric care in the Philippines remains limited and concentrated in urban tertiary hospitals. Specialized geriatric services are scarce, and community‐based screening and long‐term care systems are underdeveloped. Comparative evidence from Southeast Asia shows that countries such as Singapore and Malaysia have begun integrating structured geriatric pathways within primary care systems, while lower‐middle‐income countries such as the Philippines continue to face fragmented implementation and workforce shortages [4].

A major structural challenge in the Philippine context is the continued reliance on family‐based caregiving. Traditionally, Filipino families have provided strong intergenerational support for older adults. However, overseas Filipino worker (OFW) migration, urbanization, and changing household structures have weakened this system, leaving many older persons with reduced daily care and limited health monitoring. This creates a growing care gap that cannot be addressed by facility‐based services alone. In this context, strengthening community‐based health systems is critical. The Barangay Health Worker (BHW) system represents a uniquely Filipino and scalable platform for addressing this gap. BHWs are embedded within communities and serve as frontline providers of health promotion, disease prevention, and basic monitoring. Evidence from low‐ and middle‐income countries demonstrates that community health workers can significantly improve access to care, continuity of services, and early detection of chronic conditions when properly trained and supported [5, 6, 7].

Expanding the role of BHWs to include geriatric care is both feasible and cost‐effective. With appropriate training, BHWs can conduct basic screening for frailty, cognitive impairment, depression, medication adherence issues, and social isolation. They can also support chronic disease monitoring and strengthen referral pathways between communities and rural health units. This approach aligns with the WHO framework for integrated, people‐centered health services for aging populations [8].

To operationalize this strategy, the following policy recommendations are proposed. First, the Department of Health may integrate standardized geriatric care modules into the national BHW training curriculum. Second, local government units may establish “Healthy Aging Support Desks” within barangay health stations to serve as focal points for older adult care. Third, a measurable national target may be set to ensure that by 2030, at least one trained BHW per barangay is designated as a geriatric care focal person. Fourth, the Philippine Health Insurance Corporation (PhilHealth) may expand benefit packages to include community‐based geriatric assessment and home follow‐up services to strengthen continuity of care.

In conclusion, while the Philippines faces significant challenges related to population aging, it also possesses a strong foundation in its Barangay Health Worker system. Strengthening and expanding the geriatric functions of BHWs offers a culturally grounded, scalable, and cost‐effective strategy to address the growing health and social care needs of older Filipinos and to support the transition toward an age‐responsive health system.

## Author Contributions

The author conceptualized the article, conducted the review of relevant literature and policy documents, developed the arguments and recommendations, drafted the manuscript, and approved the final version for submission.

## Funding

The author has nothing to report.

## Conflicts of Interest

The author declares no conflicts of interest.

## Data Availability

The data supporting the findings discussed in this Letter to the Editor are publicly available from the Philippine News Agency article on the aging population in the Philippines. These data can be accessed at: https://www.pna.gov.ph/articles/1268289. No new datasets were generated during the preparation of this manuscript. The article relies on publicly available data and information obtained from cited sources.
